# The Poor Man's Home.—XXVI

**Published:** 1898-02-19

**Authors:** 


### Modern Sociology.

### THE POOR MAN'S HOME.—XXVI.

### Ticketed Rooms and Other Lodgings.

The "Family Home" which has lately been instituted in Glasgow has been so recently described in these columns that it is hardly necessary to do more than refer to it here. We may state with regret, however, that it is not a financial success. Of the class for whom it was intended many seem to resent the "institutional" character of the place, while social pride keeps from it many of a higher grade who would more thoroughly appreciate its benefits. The fault seems to be that it has been begun on too large a scale. Had an old house been arranged for the reception of a few families, the initial expenditure would have been much smaller, and the popularity of the work could have been conveniently tested. At present there are, indeed, 80 inmates in the home, but there is accommodation for 700, and many of the expenses of the establishment cannot be reduced in proportion to the number of occupants. The arrangements of the home are so comfortable, the charges made for it so small, and the need for it so obvious, that it will be a pity if the experiment proves a failure. Perhaps, even if the labouring classes avoid it, it may become popular among widows and widowers of the poorer middle class, who, as little as the working man, can afford to pay for the care of their children when they are out at work.

The corporation of Huddersfield has introduced into its lodging-house system quarters for married couples. It seems to us that this is a step of doubtful wisdom. It is better for even the poorest married couples to have a home of their own. If they cannot afford to furnish even a single room, then, we should say, they cannot afford to get married. England has long passed the stage when increased population is *ipso facto* a boon; and to make the path of marriage too easy to the very poor, who are also in many cases the improvident, is simply to encourage the birth of a more or less pauper population, and, in the end, to repeat the blunders of the old poor law—a law which, as it is now universally admitted, did most harm to the poor by the very provisions which were intended most to benefit them. Nor can one forget the conveniences such an accommodation gives for illegal relations. The manager of a lodging-house cannot invariably ask for the marriage certificate of all his lodgers. He must take their word for the tie that unites them, and who can doubt that he will often receive a false reply? It may not be the business of a corporation to interfere with the morals of the inhabitants of the town; but, at least, it should not make immorality easy. If our reasoning wrongs the good people of Huddersfield, we offer them our apologies; but we judge the possible course of events by human nature as we have found it elsewhere.

In Glasgow, the Police Act confers great powers on the municipality, which have enabled them to deal more vigorously than many other towns with the evil of overcrowding. The police can regulate the number of occupants of houses containing not more than three rooms, when the air-space of these does not exceed an aggregate capacity of 2,000 cubic feet, exclusive of lobbies and recesses. To such houses a tin ticket is affixed, stating the number of persons who may occupy the dwelling. This is calculated on a basis of 300 cubic feet per adult, two children under eight years of age being regarded as equivalent to one adult. The police have a right to inspect such ticketed tenements at any time, and a regular system of night inspection prevails. If the dwellings are found to be overcrowded the occupants are prosecuted. This method of dealing has caused decent people to leave these ticketed rooms, and the occupants are for the most part either disreputable characters or the poorest of casual labourers, in both cases people who pay their rent very irregularly. For such people not much can be done; and the ticket, which, of course, with its system of inspection, they bitterly resent, is at least so much of a blessing that it keeps them from overcrowding to an unhealthy degree Landlords, too, object to ticketing, as it drives away their best tenants. Therefore, they are always warned when it is intended to put up tickets on their property, so that they can, by removing the tenants who are guilty of the overcrowding, prevent this stigma being attached to their property. By this means landlords themselves are made more vigilant, and the development of slums is prevented. At present the number of "ticketed" dwellings in Glasgow is about 23,000, of which one-third are one-room tenements, and the remainder two-room ones. The three-room dwelling, to which the law has power to affix a ticket, has now attained a stage of decency when such regulations are no longer required.

We have now given an outline of what has been done for the housing of the poor in several large towns, from which it must seem that the authorities there are fully determined that the working man will not lack the environment needed to let him live and bring up his children in health and strength. It is in villages and small towns, especially when these date from some antiquity, that the worst conditions now prevail. Yet even in the most remote parts the impulse is spreading. Even where old buildings remain which are sanitary eyesores, any new ones that are built are adapted to those needs of health which we have not discovered until long after the needs of luxury were satisfied. There is, in fact, something of a danger that in cheap property too much may be attempted for too little money. We speak especially of plumbing and sanitary arrangements. Good plumbing is a great boon; but bad plumbing is one of the worst things possible. The old-fashioned privy, well outside the house, whatever may be its comparative inconveniences, was better than the most modern arrangements if these are not carried out so carefully as to prevent any escape of sewer-gas within the dwelling. The ideal of health in this matter would certainly be that all excreta, all domestic filth, should be disposed of outside the house. Good drainage can give much greater convenience in the disposal of waste matters, without danger to health; but bad drainage simply leaves about a house, to poison the inmates, those matters which, under an older and simpler system, would at once be conveyed elsewhere. Therefore, in places where money must be economised, it is better to make no arrangement for the disposal of slops within the house. For this reason many houses built in the early days of plumbing are worse, from a sanitary point of view, than older houses which have no plumbing at all.

